# Pathogenic adaptations to host-derived antibacterial copper

**DOI:** 10.3389/fcimb.2014.00003

**Published:** 2014-02-03

**Authors:** Kaveri S. Chaturvedi, Jeffrey P. Henderson

**Affiliations:** Division of Infectious Diseases, Department of Internal Medicine, Center for Women's Infectious Diseases Research, Washington University School of MedicineSt. Louis, MO, USA

**Keywords:** copper, pathogenesis, yersiniabactin, copper tolerance, metal biology, copper resistance

## Abstract

Recent findings suggest that both host and pathogen manipulate copper content in infected host niches during infections. In this review, we summarize recent developments that implicate copper resistance as an important determinant of bacterial fitness at the host-pathogen interface. An essential mammalian nutrient, copper cycles between copper (I) (Cu^+^) in its reduced form and copper (II) (Cu^2+^) in its oxidized form under physiologic conditions. Cu^+^ is significantly more bactericidal than Cu^2+^ due to its ability to freely penetrate bacterial membranes and inactivate intracellular iron-sulfur clusters. Copper ions can also catalyze reactive oxygen species (ROS) generation, which may further contribute to their toxicity. Transporters, chaperones, redox proteins, receptors and transcription factors and even siderophores affect copper accumulation and distribution in both pathogenic microbes and their human hosts. This review will briefly cover evidence for copper as a mammalian antibacterial effector, the possible reasons for this toxicity, and pathogenic resistance mechanisms directed against it.

## Introduction

Copper is both an essential mammalian micronutrient and a potent antibacterial agent. The Smith Papyrus, an ancient Egyptian medical text dated at 2400 BC, is the earliest medicinal archive to recommend copper sulfate to sterilize water and treat infections (Dollwet and Sorenson, 1985). Mesoamerican and Hellenistic civilizations used copper and copper salts to treat a broad variety of physical ailments, including microbial and parasitic infections. In 400 BC, Hippocrates prescribed copper salts to treat leg ulcers. In the nineteenth century, Victor Burq observed that copper workers in Paris appeared immune to recurrent cholera epidemics (Burq, 1867). He also noted that while neighboring towns were ravaged by frequent cholera epidemics, the pottery-making town of Aubagne was protected from these outbreaks. He attributed this protection to “… a rampart of copper dust” generated by copper-rich clay used by the city's potters. These observations led to rapid developments in the field of metallotherapy, and medically employed copper salts, amulets, and belts were widely used to treat dermatologic, gastrointestinal, and tubercular infections (Dollwet and Sorenson, 1985; Borkow, 2005) until the advent of commercially available antibiotics in 1932.

Human and animal studies now suggest a parallel between ancient medicinal copper use and antibacterial immune function. In this review, we summarize copper homeostasis mechanisms in the human host, and the means by which the host deploys the metal to combat infections. We describe the chemical and biochemical principles that define copper's toxicity, and how these toxic properties serve as potent leverage against invading pathogens. Finally, we discuss the pathogenic molecular, cellular, and biochemical responses that counter copper toxicity at host interface.

### Copper as nutrient or toxin

With photosynthesis and dioxygen release in the atmosphere 2.7 billion years ago, the sulfides that sequestered copper were oxidized to sulfates, leading to increased copper bioavailability (Frausto da Silva and Williams, 1993). Copper-containing proteins appeared relatively late in an evolutionary timescale, likely in response to increasing need to use oxygen and oxygen containing molecules (Dupont et al., 2011; Nevitt et al., 2012). These enzymes are critical to cellular, biochemical and regulatory functions in the human host, leading to a nutritional requirement for sufficient copper levels. The most prominent examples include cytochrome c oxidase, the respiratory chain terminal electron acceptor, and Cu-Zn superoxide dismutase, required for defense against oxidative damage (Karlin, 1993). Putative copper binding proteins compose ~1% of the total eukaryotic proteome, suggesting that known cuproproteins represent only a minor fraction of the total (Andreini et al., 2008). Copper's role in host biology and defense is better understood by examining its chemistry.

#### Copper chemistry

Copper is the 26th most abundant in the earth's crust and exists as 2 stable and 9 radioactive isotopes. A transition metal, copper primarily exists as one of two stable oxidation states: Cu^2+^ in the oxidized cupric form, and Cu^+^ in the reduced cuprous form. Cu^+^ is a closed shell 3d^10^ transition metal ion with diamagnetic properties (Frausto da Silva and Williams, 1993). A soft Lewis acid, it favors tetrahedral coordination with soft bases such as hydrides, alkyl groups, cyanide, phosphines, and thiols from cysteine and thioether bonds with methionine (Crichton and Pierre, 2001). Cu^2+^ has a 3d^9^ configuration, is paramagnetic, and is an intermediate Lewis acid. In addition to ligands bound by Cu^+^, Cu^2+^ forms square planar complexes with sulphates, nitrates, nitrogen donors such as histidine, and oxygen donors like glutamate and aspartate (Bertini et al., 2007). Different ligand combinations, oxygenation levels, pH, organic matter, sulfates and carbonates, generate differential metal speciation and distinct metal coordination environments. Copper's value as a bioelement lies mainly in its unique electrochemical properties. The Cu^+^/Cu^2+^ couple has a high redox potential, which allows it to act as an electron donor/acceptor in redox reactions (Crichton and Pierre, 2001). Most copper enzymes span a range of +200 to +800 mV, enabling them to directly oxidize substrates such as ascorbate, catechol, and phenolates. The same electrochemical properties contribute to copper's toxic effects through several mechanisms, outlined below.

#### Copper as a Fenton reagent

Within superoxide and hydrogen peroxide-rich environments such as the phagosome, copper may propagate toxic hydroxyl radical formation by Fenton-like chemistry [Equation (1)] (Liochev, 1999).

(1) $${\text{Cu}}^{+}+{\text{H}}_{2}{\text{O}}_{2}\to {\text{Cu}}^{2+}+{\text{OH}}^{-}+\text{OH}•$$

Hydroxyl radicals are extremely reactive, cannot be scavenged by enzymatic reaction, and have a diffusion controlled half-life of ~10^−9^ s before reacting with organic molecules *in vivo* (Freinbichler et al., 2011), suggesting that hydroxyl radical damage would occur in close spatial proximity to copper ions. Extensive work has implicated reactive oxygen species (ROS) derived from metal-catalyzed oxidation in lipid, protein, and DNA oxidation (Yoshida et al., 1993; Liochev, 1999; Stadtman, 2006). Copper ions can also oxidize sulfhydryls such as cysteine or glutathione in a cycle between reactions [Equations (2), (3a,b) or (4a,b), followed by (**5**)]:

(2) $$\text{RSH}+{\text{Cu}}^{2+}\to \text{RS}•+{\text{Cu}}^{+}+{\text{H}}^{+}$$

and

(3a) $$\text{RS}•+{\text{O}}_{2}\to {\text{RS}}^{+}+{\text{O}}_{2}^{-}$$

(3b) $${\text{RS}}^{+}+\text{RSH}\to \text{RSSR}+{\text{H}}^{+}$$

or

(4a) $$\text{RS}•+\text{RSH}\to \text{RSSR}+{\text{H}}^{+}$$

(4b) $$\text{RSSR}•+{\text{O}}_{2}\to \text{RSSR}+{\text{O}}_{2}^{-}$$

followed by

(5) $${\text{O}}_{2}^{-}+{\text{H}}^{+}\to 1/2{\text{H}}_{2}{\text{O}}_{2}$$

Hydrogen peroxide can in turn participate in reaction 1 and may further propagate radical formation.

Attempts to understand copper toxicity through classic copper-catalyzed Fenton chemistry to copper toxicity have produced contrary results. Macomber et al. exposed an *Escherichia coli* mutant with multiple copper efflux deficiencies to hydrogen peroxide (Macomber et al., 2007). Rather than exhibiting greater peroxide sensitivity [through Equation (1)], copper-loaded *E. coli* were instead more resistant to hydrogen peroxide. Furthermore, copper loading was associated with fewer, not more, oxidative DNA lesions. Lastly, EPR spectroscopy revealed no change in hydroxyl radical generation with copper addition. Most of the copper in overloaded strains was localized to the periplasm, where any hydroxyl radical generated would react locally before reaching DNA in the cytoplasm. This spatial compartmentalization may explain the lack of DNA damage. While there may exist circumstances in which copper propagates cytotoxic Fenton chemistry *in vivo*, this work suggests the existence of an alternative copper toxicity mechanism in *E. coli*.

#### Non-Fenton destruction of iron-sulfur complexes by copper

Recent evidence suggests a non-Fenton chemistry copper toxicity mechanism in which the reduced Cu^+^ ion is instrumental. Multiple investigators note that copper toxicity to bacteria is sustained or even enhanced in anoxic conditions (Beswick et al., 1976; Outten et al., 2001; Macomber and Imlay, 2009) where peroxide formation is minimal. Increased copper toxicity under anoxic conditions may reflect higher Cu^+^ prevalence. *E. coli* EPR spectroscopy indicates that considerable Cu^2+^ is converted to non-paramagnetic Cu^+^ under anoxic conditions (Beswick et al., 1976). Macomber et al. show that intracellular copper in overloaded *E. coli* is in the reduced Cu^+^ valence, likely due to cytosolic reduction and its ability to enter bacteria by traversing bacterial membranes (Macomber et al., 2007). Cu^+^ toxicity in the *E. coli* cytosol can be explained by its intense thiophilicity, which is sufficient to competitively disrupt key cytoplasmic iron-sulfur enzymes both *in vitro* and *in vivo* (Macomber and Imlay, 2009). Indeed, other “soft” thiophilic metal ions that do not act as Fenton reagents have been found to exert comparable toxicity (Jozefczak et al., 2012; Xu and Imlay, 2012). Together, these data provide compelling evidence linking copper toxicity to iron displacement from solvent-exposed dehydratase iron-sulfur clusters, resulting in metabolic disruption and branched chain amino acid auxotrophy.

### Copper at the host-pathogen interface

Copper homeostasis is essential for human growth and development. Average daily human dietary copper intake varies from 0.6 to 1.6 mg/dL, with a free copper ion concentration of 10^−13^ M in human blood plasma (Linder and Hazegh-Azam, 1996). In mammalian cells, cytoplasmic metallothioneins, glutathione based redox maintenance, and the Cu/Zn superoxide dismutase mitigate copper toxicity (Fridovich, 1974; Babula et al., 2012; Hatori et al., 2012). This section reviews the basic characteristics of human copper transporters together with data that may speak to their functions during infection and inflammation.

#### Human copper physiology

Unlike antimicrobial peptides, proteolytic enzymes, or ROS, copper cannot be synthesized *in situ* during infections and so must be absorbed from the diet or mobilized from tissue depots for use by immune cells (see a more complete review Pena et al., 1999). Once dietary copper is absorbed from the intestinal lumen it is delivered to the liver, which exports it to the peripheral circulation or excretes it into the bile (Crampton et al., 1965; Vancampen and Mitchell, 1965). The liver incorporates copper into multiple proteins, including the secreted glycosylated multi-copper ferroxidase ceruloplasmin (Holmberg and Laurell, 1948). Ceruloplasmin-copper complexes bind Ctr1, an integral membrane protein that is structurally and functionally conserved from yeast to humans (Zhou and Gitschier, 1997). Ctr1 transports 60–70% of the total copper in flux. Ctr1 is responsive to copper levels: copper depletion increases Ctr1 expression at the plasma membrane through the recruitment from the intracellular pools, whereas elevated copper induces rapid transporter endocytosis from the plasma membrane to vesicles (Zhou and Gitschier, 1997; Petris et al., 2003; Guo et al., 2004). Following internalization by Ctr1, copper is shuttled to the trans-Golgi network by ATOX1/HAH1 in secretory compartments (Klomp et al., 1997). Atox1 gene deletion in mice results in perinatal lethality, reflecting its crucial role in normal cellular metabolism (Hamza et al., 2001). Copper is transferred directly from ATOX1 to the N-terminus of two homologous P_1B_-type ATPase Cu^+^ transporters, ATP7A (Chelly et al., 1993; Mercer et al., 1993; Vulpe et al., 1999) and ATP7B (Bull et al., 1993; Tanzi et al., 1993; Vulpe et al., 1993), located in the trans-Golgi network. Macrophages infected with *Salmonella typhimurium* exhibit increased Ctr1, ATP7A and ceruloplasmin gene expression, indicating that they play a role in restricting infection by professional intracellular pathogens (Achard et al., 2012).

Copper fills varied roles in mammalian biology, and it is notable that copper-deficiency is associated with numerous deficiencies in host defense (Kaim and Rall, 1996). Mutations in ATP7A result in a severe copper-deficiency known as Menkes disease (Kaler, 2011). Infants with Menkes' disease are more susceptible to Gram-negative infections, consistent with copper's role in restricting microbial growth (Menkes et al., 1962; Danks et al., 1972; Gunn et al., 1984). Conversely, Wilson's disease is characterized by excess copper accumulation in brain and liver tissues, resulting in cirrhosis and neurodegeneration that may manifest well after infancy. Other human copper deficiency studies reveal impaired phagocytic indices, decreased antibody response, impaired peripheral mononuclear cell proliferation, lower early T-cell activation and proliferation, and lower cytokine expression (Sullivan and Ochs, 1978; Prohaska and Lukasewycz, 1990). While these conditions suggest a specialized role for copper in antibacterial immunity, caution must be taken to differentiate this from a less specific, more general nutritional role in the host (Newberne et al., 1968; Sullivan and Ochs, 1978; Boyne and Arthur, 1981; Jones and Suttle, 1983; Koller et al., 1987; Prohaska and Lukasewycz, 1990; Crocker et al., 1992; Smith et al., 2008).

#### Copper physiology during infections

Although incompletely understood, there are indications that a coordinated physiologic response may increase both systemic and local copper availability during infections. Compared to normal controls, copper levels increase two- to ten-fold in the serum, livers and spleens of animals infected with a range of pathogens, including viruses, bacteria, and trypanosomes (Tufft et al., 1988; Crocker et al., 1992; Matousek De Abel De La Cruz et al., 1993; Ilback et al., 2003). Increased circulating copper may be selectively imported into infected sites, as indicated by two- to five-fold increase in copper-carrier proteins (Natesha et al., 1992; Chiarla et al., 2008). X-ray microprobe analyses indicate that copper's absolute atomic concentration in area density increases a hundred-fold to several hundred micromolar within granulomatous lesions of lungs infected with *Mycobacterium tuberculosis*, and high copper concentrations are selectively redistributed to the exudates of wounds and burns (Beveridge et al., 1985; Jones et al., 2001; Voruganti et al., 2005; Wagner et al., 2005). Whether this accumulation reflects uptake by myeloid cells alone or includes a tissue-wide response remains unclear.

#### Copper as a white blood cell antibacterial agent

In 2009, White et al. published findings from cultured macrophage-like RAW264.7 cells that are consistent with a copper-specific bactericidal system directed against phagocytosed *E. coli* (White et al., 2009). Phagosomal killing of K12 *E. coli* was greatly affected by copper content of the cell culture media. Microscopy and posttranscriptional silencing investigations linked this copper-dependent activity to ATP7A-mediated copper trafficking from the Golgi apparatus to *E. coli*-containing phagolysosomes. These studies suggest that in addition to its role in physiologic copper absorption, ATP7A fills a host defense function by transporting antibacterial quantities of copper ions to phagolysosomal compartments containing engulfed bacteria. Consistent with this finding, low-density lipoprotein (LDL) oxidation by macrophage-like THP-1 cells was found to be ATP7A-dependent, suggesting metal catalyzed oxidation by secreted copper ions (Qin et al., 2010). ATP7A is expressed in a broad range of both myeloid and non-myeloid cell types (La Fontaine et al., 2010; Wang et al., 2011), raising the possibility that a variety of cell types may similarly direct the copper payloads to kill internalized bacteria. These observations suggest a specific functional rationale for the array of mammalian copper transport genes upregulated by proinflammatory stimuli such as interferon-gamma and lipopolysaccharide and for the altered copper physiology noted above in section Copper as a Fenton Reagent. (Achard et al., 2012). Studies to identify macrophage lineages or even non-professional phagocytes that use copper-mediated antibacterial activity would be of great interest in the area of infection biology. To date, copper-dependent uropathogenic *E. coli* killing has been observed in both RAW264.7 cells and mouse peritoneal macrophages (Chaturvedi et al., 2013). Altogether, these findings suggest an intriguing parallel between ancient medicinal copper use and innate immune function.

Phagosomal copper may add to, and perhaps synergize with, the diverse cellular microbial killing strategies described since Elie Metchnikoff's pioneering work on phagocytosis (Gordon, 2008). These strategies are often functionally redundant and have been broadly grouped into oxidative killing mechanisms exemplified by the macrophage respiratory burst and non-oxidative killing mechanisms such as antimicrobial peptides and hydrolytic enzymes. Interactions between copper and more established antibacterial effectors within the phagosome's restricted space are likely. Membrane permeabilizing defenses may facilitate copper entry into bacteria, while high concentrations of respiratory burst-derived oxidants are likely to modulate redox active copper ions. These interactions may be spatially and temporally governed during and after the respiratory burst. One recent finding in *E. coli* suggests that copper's interactions with phagosomal superoxide may greatly impact intracellular bacterial survival (see section Superoxide Dismutation).

Copper-mediated killing by vertebrate immune systems would be expected exert selective pressure on copper resistance in pathogenic bacteria. Below, we review the virulence-associated copper resistance systems described in several human pathogens. The classic intracellular pathogen *M. tuberculosis* upregulates genes encoding copper efflux-associated P_1B_-type ATPases during macrophage infection (Ward et al., 2008; Rowland and Niederweis, 2012). Urinary *E. coli* isolates collected from patients with urinary tract infections (UTIs) exhibit higher growth than concomitant rectal isolates in a medium containing an inhibitory concentration of copper (Chaturvedi et al., 2012). Copper resistance genes are often observed in virulence-associated mobile genetic elements carried by *E. coli* as well as *Legionella pneumophila*, *Klebsiella pneumoniae*, and methicillin resistant *Staphylococcus aureus* (Sandegren et al., 2012; Shoeb et al., 2012; Gomez-Sanz et al., 2013; Trigui et al., 2013). *E. coli* and *M. tuberculosis* strains with engineered deficiencies in copper resistance genes exhibit impaired intracellular survival in phagocytic cells (White et al., 2009; Wolschendorf et al., 2011; Chaturvedi et al., 2013). To date, these observations suggest that resistance to copper-mediated killing among pathogens may be a virulence-associated property driven by host innate immunity.

### Mechanisms of microbial copper tolerance

Copper's direct and indirect toxicity can alter enzyme specificity, disrupt cellular functions, and damage nucleic acid structure. Changes in copper concentrations during infection suggest that the host harnesses the metal's toxic properties to combat microbial growth. In response, pathogenic bacteria have evolved a series of protein- and small-molecule based defenses against copper toxicity. Unlike eukaryotic cells, most known bacterial cuproproteins are located within the cytoplasmic membrane or in the periplasmic space, perhaps to compartmentalize a potentially toxic metal species. Microbes use this copper sparingly in metabolism, and for electron transport in respiratory pathways. Given this, copper's cytoplasmic availability is tightly controlled, and data indicate that there are fewer than 10^4^ free copper atoms per bacterial cell, reflecting cytoplasmic copper-responsive transcriptional regulators' high copper sensitivity (Outten and O'Halloran, 2001; Changela et al., 2003; Finney and O'Halloran, 2003).

Both Cu^+^ and Cu^2+^ can permeate the outer membrane of *E. coli* and enter the periplasm, but only Cu^+^ is able to cross the inner membrane and reaches the cytoplasm by a currently unknown mechanism. While no copper uptake genes have yet been identified in *E. coli*, the outer-membrane protein ComC (under transcriptional control of the TetR-like regulator ComR) may reduce the outer membrane's copper permeability (Mermod et al., 2012). It is speculated that cytoplasmic Cu^+^ is largely complexed by millimolar quantities of thiols such as glutathione. Interestingly, glutathione biosynthesis gene deletion has little effect on microbial copper response, indicating that its role in detoxifying copper in bacterial cells may either be limited or redundant (Helbig et al., 2008). In this regard, qualitative and quantitative analyses of cytosolic copper binding sites in bacteria would aid our understanding of copper toxicity.

**Figure 1:**
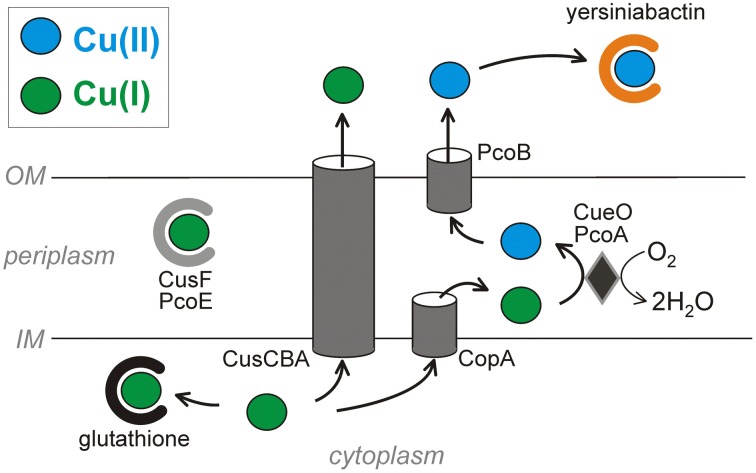
**Copper resistance strategies across pathogenic *E. coli* membranes**. The virulence-associated siderophore yersiniabactin sequesters Cu^2+^ outside the cell and prevents its reduction to the more toxic Cu^+^. Copper ions that reach the cytosol are subject to chelation by glutathione and export by two ATPases. The CusCBA ATPase complex exports Cu^+^ from both the cytoplasm and the periplasm (*via* CusF) to the extracellular space. Alternatively, the CopA ATPase exports cytoplasmic copper across the inner membrane. Periplasmic Cu^+^ can bind the proteins CusF and PcoE or be oxidized by the mixed copper oxidases CueO or PcoA to less toxic Cu^2+^. PcoB has a putative function of exporting Cu^2+^ across the outer membrane. The systems are oriented to minimize free cytosolic copper ions by directing these to the periplasmic or extracellular spaces.

Microbial copper-resistance systems span copper efflux (*cue*, *cus*, and extrachromosomal efflux systems), copper sequestration (CusF and siderophores), and copper oxidation (mixed copper oxidases and superoxide dismutase mimics). For the sake of brevity, the following sections primarily discuss Cu^2+^ detection and resistance proteins that have been described in *E. coli* (Figure 1). Their functional homologs in other microbial species are tabulated in Table 1 (see a more complete review Rademacher and Masepohl, 2012).

**Table 1:** **Species-wide distribution of copper-resistance proteins**.

**Function**	**Protein**	**Species**
Copper detection	SctR GolS^*^	*S. typhimurium* (Espariz et al., 2007; Pontel et al., 2007; Osman and Cavet, 2011)
	RicR	*M. tuberculosis* (Festa et al., 2011)
	CueP	*S. typhimurium* (Pontel and Soncini, 2009)
	CrdRS	*H. pylori* (Waidner et al., 2005)
	CopY	*Enterococcus hirae, Enterococcus faecium, Streptococcus mutans, Lactococcus lactis* (Strausak and Solioz, 1997; Vats and Lee, 2001; Magnani et al., 2008; Reyes-Jara et al., 2010)
Copper efflux	GolT	*S. typhimurium* (Espariz et al., 2007; Osman et al., 2010)
	CtpV	*M. tuberculosis* (Rowland and Niederweis, 2012)
	CopA1	*P. aeruginosa* (Thaden et al., 2010)
	CopA2	*P. aeruginosa* (Gonzalez-Guerrero et al., 2010)
	CopA	*Enterococcus hirae* (Odermatt et al., 1994; Odermatt and Solioz, 1995)
	CopB	*Enterococcus hirae* (Odermatt et al., 1994; Odermatt and Solioz, 1995)
Copper sequestration	CueP	*S. typhimurium* (Pontel and Soncini, 2009)
	SmtA	*Synechococcus* (Shi et al., 1992)
Copper oxidation	Rv0846c	*M. tuberculosis* (Rowland and Niederweis, 2013)
Copper homeostasis repression	YcnK	*Bacillus subtilis* (Chillappagari et al., 2009)
	CstR	*Staphylococcus aureus* (Grossoehme et al., 2011)

* *Confers additional protection from gold toxicity (Espariz et al., 2007; Pontel et al., 2007)*.

#### Copper efflux

***The cue system***. In *E. coli*, two chromosomal systems remove excess Cu^+^ from the cytosol (Outten et al., 2001). The *cue* system (for *Cu e*fflux) transcriptionally activates both plasmid- and chromosomally-encoded copper homeostatic systems in response to intracellular Cu^+^ sensing through CueR, a MerR-family metalloregulatory transcriptional activator (Petersen and Moller, 2000; Stoyanov et al., 2001). CueR coordinates one Cu^+^ ion per monomer in an unusual and distinctive linear S–Cu^+^–S center encompassing two cysteine residues (C112 and C120) located at the dimer interface (Changela et al., 2003; Chen et al., 2003). Both *holo*- and *apo*-CueR bind to dyad-symmetric sequences at target promoters, but only *holo*-CueR activates transcription (Yamamoto and Ishihama, 2005; Andoy et al., 2009). A genome-wide transcriptional array study of the *E. coli* chromosome has identified 197 putative CueR-binding sites, which largely await experimental confirmation. Other bacteria that possess CueR-like copper-tolerance systems include *Pseudomonas aeruginosa* and *S. typhimurium* (Espariz et al., 2007; Pontel and Soncini, 2009; Thaden et al., 2010).

CueR is a copper-selective ortholog from multifunctional protein families that respond to a wide range of effector ligands (the MecI/BlaI-family repressors that mediate resistance to β-lactam antibiotics and the MerR family, respectively) (Brown et al., 2003; Portmann et al., 2006). While CueR is not widely distributed in bacterial genomes, Liu et al. describe one such copper-specific ubiquitous regulator (Liu et al., 2007). The intracellular copper sensor CsoR from *M. tuberculosis* is the founding member of what appears to be a large family of bacterial Cu^+^-responsive repressors, with greater than 170 projected members in archaeal, bacterial, and cyanobacterial genomes (Liu et al., 2007). Upon copper binding, CsoR is deactivated, leading to copper-resistance gene expression.

CueR upregulates *copA* and *cueO* gene expression (Outten et al., 2000; Stoyanov et al., 2001). These genes are associated with copper efflux and oxidation, respectively. CopA is a copper-exporting P_1B_-type ATPase active under high extracellular copper stress (Outten et al., 2000; Petersen and Moller, 2000; Fan and Rosen, 2002; Stoyanov et al., 2003). Mammalian and microbial P_1B_-type ATPases thus perform opposing functions that determine infection outcomes. Appropriate copper import and trafficking by mammalian ATPases is required to restrict microbial growth, while copper export by microbial ATPases is necessary to withstand this toxicity. CopA traverses the inner membrane and exports Cu^+^ from the cytosol in both oxic and anoxic conditions (Fan and Rosen, 2002; Kuhlbrandt, 2004; Arguello et al., 2007; Osman and Cavet, 2008). This efflux pump couples ATP hydrolysis to form an acylphosphate intermediate in the presence of Cu^+^ but not Cu^2+^. It is speculated that two amino-terminal metal binding domains with a CXXC motif confer metal binding specificity. *copA* mutants in *E. coli*, *Streptococcus pneumoniae*, and *Neisseria gonorrhoeae* all demonstrate impaired copper efflux, intracellular metal accumulation, and increased copper sensitivity in both oxic and anoxic conditions (Rensing et al., 2000; Outten et al., 2001; Shafeeq et al., 2011; Djoko et al., 2012).

***The cus system***. An independent copper efflux system, the *cus* (for *Cus* ensing) system confers copper-tolerance under moderate to high copper levels in oxic conditions (Outten et al., 2001). *cusRSCFBA* products are believed to form a multiunit transport complex that spans the periplasmic space and is anchored in both the inner and outer membranes (Mealman et al., 2012). While CopA exports excess Cu^+^ from the cytoplasm to the periplasm, CusRSCFBA effluxes Cu^+^ from the periplasm (Outten et al., 2001; Franke et al., 2003; Long et al., 2010).

CusRS is a two-component regulatory system that monitors copper stress in the cell envelope and is particularly active in anoxic copper stress conditions (Munson et al., 2000). In addition to CusRS, CpxRA, and YedWV are two other previously described copper-responsive *E. coli* two-component regulatory systems (Yamamoto and Ishihama, 2005, 2006). CusR and CusS exhibit homology with other plasmid-borne two-component systems that are also involved in metal responsive gene regulation. Membrane bound CusS senses periplasmic Cu^+^, which leads to protein autophosphorylation. CusS then donates the phosphoryl group to CusR, which activates the transcription of the *cusCFBA* and *cusRS* operons. CusA belongs to the resistance-nodulation-cell division (RND) proton antiporter family, CusB belongs to the membrane fusion protein family which anchor into the cytoplasmic membrane with a long periplasm-spanning domain, and CusC is an outermembrane protein with homology to the TolC-stress response protein (Franke et al., 2003; Delmar et al., 2013). CusF is a periplasmic metallochaperone that binds a single atom of Cu^+^ and participates in metal efflux by delivering the metal to CusC and CusB (Xue et al., 2008; Mealman et al., 2011).

Other prominent RND proton antiporters include the multidrug efflux systems AcrB and AcrF from *E. coli*, MexB from *P. aeruginosa*, and MtrD from *N. gonorrhoeae* (Nies and Silver, 1995; Paulsen et al., 1996). Interestingly, *Cupriavidus metallidurans* CH34 resistance to copper is attributed to RND protein expression (von Rozycki and Nies, 2009).

***Extrachromosomally-encoded copper efflux systems***. In environments where copper concentrations would overwhelm chromosomally encoded copper metabolic systems, microbes contain extrachromosomal loci that confer copper resistance. These loci are present in copper-resistant *E. coli*, *Pseudomonas syringae*, and *Xanthomonas campestris* pv. vesicatoria isolates (Tetaz and Luke, 1983; Bender and Cooksey, 1987; Brown et al., 1992; Voloudakis et al., 1993; Williams et al., 1993). All copper-resistant strains were isolated from agricultural areas characterized by repeated copper salt application as a feed additive, bactericidal agent, or antifungal agent. In these strains, the plasmid borne *pco* and *cop* operons confer copper resistance. These operons carry four related genes, *pcoABCDRSE* and *copABCDRS*, which are expressed from chromosomal copper-inducible promoters regulated by CusRS (Brown et al., 1995; Adaikkalam and Swarup, 2005). The genes *copABCDRS* are arranged in two operons, *copABCD* and *copRS*, respectively. This arrangement is also found in the *pco* determinant but with an additional gene, *pcoE*, further downstream. Extrachromosomal systems encode two-component regulators similar to CusRS, including PcoR and PcoS from the *pco* operon of *E. coli*; CopR and CopS from the *cop* operon, which provides copper resistance to *P. syringae*; and SilR and SilS from the *sil* locus, which provides silver ion resistance to *Salmonella enterica* serovar Typhimurium (Gupta et al., 1999). Similar to these copper efflux systems, extrachromosomal *pco* system encodes PcoB and PcoD, two copper pumps that are incorporated in the outer and inner membranes, respectively (Lee et al., 2002).

Extrachromosomal resistance systems are metal oxidation state selective. Recently published PcoC spectroscopic and crystallographic data and nuclear magnetic resonance (NMR) studies of the closely related *P. syringae* protein, CopC, reveal a biologically unprecedented thioether ligation (Arnesano et al., 2003a,b; Peariso et al., 2003). PcoC can bind both Cu^2+^ and Cu^+^: the protein exhibits a cupredoxin fold that binds Cu^+^ through two Met sulfur atoms and one nitrogen or oxygen ligand in a hydrophobic Metrich loop that is exposed to solvent on the protein surface. Cu^2+^ can bind a separate site in the same protein, where it coordinates water, as well as two histidine imidazoles and two other nitrogen or oxygen ligands. Following copper sensing, microbes respond to microenvironments that contain high concentrations of unligated copper by upregulating systems associated with copper efflux, oxidation, or sequestration.

#### Copper sequestration

In addition to copper oxidation and efflux systems, recent studies suggest that bacteria deploy both low molecular weight proteins and small molecules to bind and sequester intracellular copper. In *E. coli*, the periplasmic chaperone CusF binds copper, ultimately delivering it to CusCBA for export (Franke et al., 2003; Bagai et al., 2008; Xue et al., 2008; Mealman et al., 2012). Evidence indicates that PcoE acts as a soluble copper binder in the periplasm (Zimmermann et al., 2012). Across kingdoms, metallotheioneines sequester cytoplasmic copper (Leszczyszyn et al., 2011; Thirumoorthy et al., 2011; Gumulec et al., 2012). Recent work in *M. tuberculosis* shows that a five-locus regulon for copper resistance is upregulated during copper stress (Festa et al., 2011). This regulon includes MymT, a cytoplasmic metallothionein that binds Cu^+^ and attenuates copper toxicity (Gold et al., 2008). Although a native *E. coli* metallothionein has not yet been identified, data suggest that glutathione may exert similar cytoprotective effects by forming stable Cu^+^ complexes (Osterberg et al., 1979; Helbig et al., 2008; Macomber and Imlay, 2009).

Some microbial siderophores, low-molecular-weight iron chelating agents, sequester copper extracellularly and protect bacteria by minimizing intracellular copper penetration. There is precedent for this among environmental bacteria that express Cu^+^-binding compounds (those originally identified as copper binders are called chalkophores) such as methanobactin and phytochelatin (Cervantes and Gutierrez-Corona, 1994; Rauser, 1999; Kenney and Rosenzweig, 2012). In *E. coli*, chemically distinct siderophore types are observed to exert opposing copper phenotypes. Specifically, the catecholate siderophore enterobactin sensitizes *E. coli* to copper, likely through its ability to reduce cupric ion to the more toxic cuprous ion (Grass et al., 2004). Although known as a cuprous oxidase, CueO prevents this interaction by directly oxidizing catechols such as dihydroxybenzoic acid, an enterobactin biosynthetic precursor (Grass et al., 2004). Conversely, phenolate siderophores such as yersiniabactin bind Cu^2+^ in complexes that prevent reductive free Cu^+^ release (Chaturvedi et al., 2012). Uropathogenic *E. coli* strains that express yersiniabactin are protected from copper's toxic effects, suggesting that a strain's small molecule repertoire may affect its ability to survive and persist in a copper-rich environment. It is notable that yersiniabactin can protect bacteria with and without FyuA (the outer membrane ferric yersiniabactin importer) from copper toxicity, suggesting that yersiniabactin's iron uptake function does not contribute to this phenotype. Copper oxidation state selectivity among microbial small molecules is also observed in pyoverdin and pyochelin, two major siderophore types expressed by *P. aeruginosa* (Brandel et al., 2012). While both siderophores can bind Cu^2+^, Cu^2+^ supplementation upregulates genes involved in the synthesis of pyoverdin but downregulates those for pyochelin (Frangipani et al., 2008; Brandel et al., 2012). Data indicate that both siderophores prevent Cu^2+^ accumulation in the bacterial cell by 80% (Teitzel et al., 2006). Pyoverdin's selective expression indicates that it may play a direct role in copper tolerance, possibly by sequestering copper in reduction-resistant complexes like yersiniabactin. The chemical basis of pyoverdin's transcriptional selectivity is unclear, and response regulation is unknown. It is possible that ferric- and cupric siderophore complexes govern differential transcriptional responses.

It also remains unclear whether siderophore transport systems can discriminate between different metal bound forms. While sequestration by siderophores can attenuate copper toxicity, bacterial proteins that import siderophore-metal complexes may also play a role. The siderophore schizokinen eliminates copper's toxic effects on *Anabaena* (Clarke et al., 1987) but exacerbates copper toxicity in *Bacillus megaterium* (Arceneaux et al., 1984). It is possible that these differences arise from fundamental differences in metabolic and transport machinery between the two organisms. Copper schizokinen-mediated toxicity in *Bacillus* can be alleviated by the exogenous desferrioxamine, raising the possibility that cells transport iron to repair copper-mediated damage. This observation could be further explained by differences in each organism's ability to use its iron-uptake machinery to discriminate between cupric- and ferric-siderophore complexes. It is possible that copper indirectly affects siderophore expression by competitively inhibiting iron import or liberating intracellular iron, altering intracellular metal accumulation, and affecting a downstream biosynthetic feedback loop.

#### Copper oxidation

***Mixed copper oxidases (MCO)***. Cu^+^ is more toxic than Cu^2+^ when applied under anoxic conditions, as demonstrated by Macomber and Imlay (2009). Consistent with this observation, *E. coli* cultures treated with both Cu^2+^ and reductants such as ascorbate or catechols demonstrate lower viability than those treated with Cu^2+^ alone (Chaturvedi et al., 2012). To detoxify extracytoplasmic Cu^+^, *E. coli* use the CueR-regulated multi-copper oxidase CueO to oxidize toxic cuprous copper to its less toxic cupric form (Grass and Rensing, 2001; Roberts et al., 2002; Singh et al., 2004). *E. coli* and *S. typhimurium* mutants lacking CueO exhibit extreme copper sensitivity in oxic conditions. CueO contributes to *S. typhimurium* virulence in a systemic murine infection model (Achard et al., 2010). A second, plasmid-borne 605 amino acid MCO called PcoA has also been described in *E. coli*. Periplasmic extracts containing PcoA exhibit copper-inducible oxidase activity, indicating that PcoA might similarly oxidize Cu^+^ to prevent toxicity (Huffman et al., 2002; Djoko et al., 2008). PcoA can functionally substitute for CueO in *E. coli*, indicating that these proteins have redundant function.

*E. coli* CueO is among the best-characterized bacterial multicopper oxidases (MCOs). CueO is structurally similar to the large, cross-Kingdom family of MCOs [including ascorbate oxidase and the ferroxidases Fet3 and ceruloplasmin (Outten et al., 2000)] that oxidize substrates using oxidizing equivalents in molecular oxygen. This oxygen requirement renders oxidases inactive under anoxic conditions. CueO's active site consists of a trinuclear copper center MCO active site in which a fourth copper atom mediates electron transfer from the substrate (Roberts et al., 2002; Grass et al., 2004). The enzyme couples Cu^+^ oxidation with four-electron oxygen oxidation to water through the hydroxide-bridged fourth copper atom. Reactive oxygen intermediates generated during the reaction remain coordinated and are not released from the protein. It is curious that despite low cytoplasmic copper levels, CueO and PcoA exhibit a twin-arginine motif in their leader sequences, suggesting that they are translocated from the cytoplasm by the twin arginine translocation (Tat) pathway with copper-bound active sites (Huffman et al., 2002). *Holo-*protein translocation from the cytoplasm means that some amount of chaperone-bound copper must be delivered to these *apo*-proteins intracellularly. This indicates that intracellular copper may serve a biosynthetic role in this specific process. If MCOs ultimately evolved to prevent copper entry to the cytosol, it is possible that metallation by cytosolic copper is a form of feedback regulation in which higher cytosolic copper levels lead to higher MCO secretion. Further studies are necessary to discern this, and other, possibilities.

In addition to oxidizing periplasmic Cu^+^, *E. coli* CueO can also oxidize 2,3 dihydrobenzoic acid (DHB) (Grass et al., 2004). 2,3-DHB is the biosynthetic precursor to enterobactin, a catecholate siderophore, secreted during iron limitation. As enterobactin can reduce Cu^2+^ to Cu^+^, it has been hypothesized that CueO's 2,3-DHB oxidation activity is a strategy to prevent toxic Cu^+^ accumulation. While it may seem paradoxical to both synthesize and destroy a siderophore, an intracellular copper requirement for CueO secretion may ensure that it's siderophore destructive activity is only relevant in the presence of high copper levels. Together, these findings suggest that MCO's such as CueO help protect bacteria from copper stress by controlling copper ion oxidation states in oxic environments.

***Superoxide dismutation***. Recent work shows that yersiniabactin expression greatly facilitates pathogen survival within phagocytic cells in a copper- and NADPH oxidase system-dependent manner (Chaturvedi et al., 2013). In the presence of copper- and NADPH oxidase-derived superoxide, yersiniabactin production protects urinary pathogenic *E. coli* within cultured macrophage-like cell phagosomes. Superoxide's contribution to this phenotype suggests that yersiniabactin's cytoprotective effects may not be attributable to copper sequestration alone. Subsequent biochemical characterizations reveal that the copper-yersiniabactin complexes catalyze superoxide dismutation according to [Equations (6) and (7)]:

(6) $${\text{O}}_{2}{•}^{-}+{\text{Cu}}^{2+}-\text{Ybt}\to {\text{Cu}}^{+}-\text{Ybt}+{\text{O}}_{2}$$

(7) $${\text{O}}_{2}{•}^{-}+{\text{Cu}}^{+}-\text{Ybt}+{\text{2H}}^{+}\to {\text{Cu}}^{2+}-\text{Ybt}+{\text{H}}_{2}{\text{O}}_{2}$$

Copper-yersiniabactin confined within the phagolysosome may thus greatly diminish concentrations of superoxide (a reductant), while maintaining or increasing production of hydrogen peroxide (an oxidant). This may have the effect of minimizing reduced Cu^+^ concentrations while increasing oxidized—and less toxic—Cu^2+^ ion concentrations. Periplasmic Cu,Zn-SOD may similarly protect against copper stress, although there are distinctive pathogenic advantages to deploying a non-protein catalyst such as copper-yersiniabactin in the phagosomal microenvironment (Chaturvedi et al., 2013). Yersiniabactin may synergize with CueO and other mixed copper oxidases by binding Cu^2+^ product ions generated by these enzymes to form catalytic copper-yersiniabactin. While interactions such as these will require further experimental validation, they fit with an overall paradigm in which pathogens appear able to convert host-supplied copper into catalysts (mixed copper oxidases, copper-yersiniabactin, Cu,Zn-SOD) that help resist copper toxicity. SOD activity may promote bacterial survival in several pathologically important host niches and its connection with copper suggests new insights into host defense mechanisms that are critical to infection pathogenesis.

## Prospects

Much remains to be understood about the mechanisms by which mammalian hosts deploy copper to resist infection, and how pathogenic bacteria respond to these strategies. ATP7A's emerging role in direct antibacterial immunity warrants its detailed study in mammalian cells that encounter bacterial pathogens. Cell type, pathogen, and regulatory activity may result in unforeseen interactions between copper and other innate immune effector molecules. Possible cooperation with mammalian copper absorption and trafficking may suggest routes by which copper-based immunity could be therapeutically supported. Both basic and translational research efforts will be necessary to understand these details.

The mechanisms by which pathogenic bacteria resist copper during mammalian infections merits further investigation. Studies conducted in bacterial cultures with environmental and pathogenic isolates provide an excellent starting point for infection models that may provide additional insights. The recent finding that yersiniabactin, a virulence-associated siderophore in *E. coli* binds copper during humans infections (Chaturvedi et al., 2012) and promotes microbial survival in phagocytic cells suggests that host microenvironments may reveal new copper resistance strategies (Chaturvedi et al., 2013). Yersiniabactin exemplifies the rich array of microbial secondary compounds that may include other copper-detoxifying microbial products. Metabolomic approaches, which are sensitive to the end products of multi-gene biosynthetic units, are well suited to discover additional copper-binding secondary compounds.

Copper's inherent toxicity has renewed interest in its use as an antimicrobial. Three hundred different copper and copper alloy surfaces are registered with the U.S. Environmental Protection Agency as antimicrobials and trials are underway to determine whether copper treated surfaces can significantly reduce nosocomial infections (http://www.epa.gov/pesticides/factsheets/copper-alloy-products.htm) (Grass et al., 2011). While these approaches may be useful in limiting nosocomial infections, it is worth noting that environmental copper-resistance loci have been isolated from Gram-negative bacteria that colonize agricultural areas repeatedly treated with copper salts. Given the linkage between copper resistance and virulence, it would be worth knowing whether sublethal copper exposures might effectively select for increased virulence in bacteria. Improved insight into bacterial copper resistance mechanisms *in vivo* and in environmental settings will be necessary to optimize antimicrobial uses of copper.

### Conflict of interest statement

The authors declare that the research was conducted in the absence of any commercial or financial relationships that could be construed as a potential conflict of interest.
